# Evaluation of agreement between invasive and non-invasive blood pressure measurement using the PetMAP^™^ device in rabbits

**DOI:** 10.3389/fvets.2023.1141480

**Published:** 2023-07-10

**Authors:** Jerneja Sredenšek, Jurij Žel, Attilio Rocchi, Nina Gasparik-Küls

**Affiliations:** ^1^University Clinic for Companion Animals, Clinical Unit of Anaesthesiology and Perioperative Intensive-Care Medicine, University of Veterinary Medicine Vienna, Vienna, Austria; ^2^Small Animal Clinic, Veterinary Faculty, University of Ljubljana, Ljubljana, Slovenia

**Keywords:** anaesthesia, agreement, blood pressure, oscillometric, rabbits

## Abstract

**Background:**

Measurement of arterial blood pressure is recommended in anaesthetized animals to guide perioperative treatment. Invasive blood pressure measurement is considered the gold standard, however it is also technically challenging, requires specialised equipment and carries certain risks. For these reasons, non-invasive blood pressure measurement devices are commonly used and are expected to provide accurate and reliable results. This requirement is particularly true for rabbits, in whom peri-anaesthetic hypotension is commonly observed and in whom perioperative mortality remains disproportionally high. Several authors have compared different non-invasive devices with invasive measurements in rabbits and have reported contrasting results. However, to date no comparison between invasive measurements and the PetMAP^™^ device, that has been designed specifically for veterinary medicine, has been reported.

**Aim and hypothesis:**

The aim of the study was the comparison of invasive blood pressure measurement with PetMAP^™^ in rabbits. We hypothesised that PetMAP^™^ would show acceptable agreement with the invasive measurements according to the American College of Veterinary Internal Medicine guidelines.

**Materials and methods:**

Sixteen client-owned rabbits presenting for various surgical interventions were included in the study. Invasive measurements were performed by cannulation of an auricular artery. The PetMAP^™^ cuff was applied distal to the elbow according to the manufacturer’s guidelines. For each measurement with PetMAP^™^, three invasive blood pressure values were recorded. The mean of the three invasive values was compared with one value measured with PetMAP^™^.

**Results:**

Data collected from 16 rabbits were used for statistical analysis. In the clinical setting, the PetMAP^™^ device showed significant overestimation of systolic, diastolic and mean arterial pressure, which were measured in the auricular artery. In addition, the bias was not constant, implying that the device poorly predicted changes in blood pressure.

**Conclusion:**

The PetMAP^™^ device did not meet any of the American College of Veterinary Internal Medicine recommendations.

## 1. Introduction

Measurement of arterial blood pressure (ABP) is considered the basic standard of anaesthesia in humans (1) and is also recommended in anaesthetized animals (2). The values measured by the blood pressure devices are used by anaesthetists to assess the patient’s cardiovascular stability, so their accuracy and reliability are of great importance.

Invasive blood pressure (IBP) measurement is the “gold standard” for measuring ABP and is also used in the validation of non-invasive blood pressure (NIBP) measuring devices (3, 4). While IBP provides accurate, »beat-to-beat« information, it requires the insertion of a catheter into an accessible artery. This can be technically challenging, requires specialised equipment (5), and also carries risks such as infection, bleeding, hematoma, embolus formation, and partial or complete occlusion of an artery (5). For these reasons alternatives such as NIBP measurement techniques are often preferred. While non-invasive and easy to use, NIBP measurement techniques may not be as accurate as IBP measurements, especially in animals (3).

To ensure accuracy of measurements, the American College of Veterinary Internal Medicine (ACVIM) has issued a consensus statement recommending validation of NIBP measurement devices (3).

PetMAP^™^ is an oscillometric ABP measuring device that was designed specifically for veterinary medicine. It features species and cuff position optimizations (6) that are believed to provide greater accuracy in ABP measurement.

PetMAP^™^ was previously tested in sheep (7), dogs (8), and cats (9). The device did not fully comply with ACVIM/Association for the Advancement of Medical Instrumentation (AAMI) recommendations in any of the species, but showed better agreement with the invasive technique in measuring mean arterial pressure (MAP). In cats, the monitor provided an acceptable trending ability for systolic arterial pressure (SAP), diastolic arterial pressure (DAP), and MAP. This study evaluates the agreement between IBP measurements and the NIBP measurements performed with PetMAP^™^ in rabbits, with the hypothesis that PetMAP^™^ shows acceptable agreement with the IBP measurements.

## 2. Materials and methods

Sixteen client-owned rabbits (six males and ten females) aged 41 +/− 19 months were included in the study. The median weight was 1.9 kg (minimum 1.3 kg and maximum 6.3 kg). Four rabbits were presented for ovarihysterectomies and 12 for dental procedures (Table 1). Gradation from ASA 1 (a normal healthy patient) to ASA 4 (a patient with severe systemic disease posing a constant threat to life) according to the American Society of Anesthesiologists physical status classification was performed. The study was discussed and approved by the institutional ethics and animal welfare committee (ETK-89/05/2019) in accordance with Good Scientific Practice guidelines and national legislation, and informed consent was obtained from the client.

**Table 1:** Anesthesia premedication, body position, complications and procedures in rabbits that were included in the study.

Case	Anaesthesia protocol – premedication	Body position	Complications	Procedure
1	Methadone 0.5 mg/kg, Medetomidine 60 mcg/kg, Ketamine 15 mg/kg IM	S, SR		Dental repair
2	Butorphanol 0.5 mg/kg, Midazolam 0.5 mg/kg intranasally	SR		Dental repair
3	Butorphanol 0.5 mg/kg, Midazolam 0.5 mg/kg intranasally	S		Dental repair
4	Methadone 0.5 mg/kg, Medetomidine 50 mcg/kg, Midazolam 0.5 mg/kg IM	D, L		OVH
5			Excluded – arterial line not possible to place	
6	Methadone 0.5 mg/kg, Midazolam 0.5 mg/kg, Ketamine 7 mg/kg IM	S		Dental repair
7	Methadone 0.5 mg/kg, Medetomidine 30 mcg/kg, Midazolam 0.5 mg/kg IM	S, D		Dental repair
8	Butorphanol 0.5 mg/kg, Medetomidine 30 mcg/kg, Midazolam 0.5 mg/kg IM	D		OVH
9	Butorphanol 0.5 mg/kg, Medetomidine 30 mcg/kg, Midazolam 0.5 mg/kg IM	S, L		Dental repair
10	Methadone 0.5 mg/kg, Medetomidine 50 mcg/kg, Midazolam 0.5 mg/kg IM	S	Euthanized during the procedure due to poor prognosis	Dental repair
11	Butorphanol 0.5 mg/kg, Midazolam 0.5 mg/kg intranasally	D		OVH
12	Methadone 0.5 mg/kg, Medetomidine 50 mcg/kg, Midazolam 0.5 mg/kg IM	S		Dental repair
13	Butorphanol 0.5 mg/kg, Midazolam 0.5 mg/kg intranasally	L, S		Dental repair – abscess
14	Methadone 0.5 mg/kg, Medetomidine 50 mcg/kg, Midazolam 0.5 mg/kg IM	D, S		Dental repair
15	Butorphanol 0.5 mg/kg, Midazolam 0.5 mg/kg intranasally	SR		Dental repair
16			Excluded – life threatning complications after anaesthesia induction	
17	Methadone 0.5 mg/kg, Medetomidine 50 mcg/kg, Midazolam 0.5 mg/kg IM	D, S		OVH
18	Butorphanol 0.5 mg/kg, Midazolam 0.5 mg/kg intranasally	SR, S		Dental repair

IM, Intramuscularly; S, Sternal; SR, Sternal raised (front part of the body raised); D, Dorsal; L, Lateral; OVH, Ovarihysterectomy.

### 2.1. Anaesthesia

A preanaesthetic examination and basic blood tests were performed in all rabbits. Based on the findings, an appropriate anaesthetic protocol was selected by an experienced anaesthesiologist. Drugs used for premedication included methadone (Methadon Streuli^®^, Streuli Pharma AG, Switzerland), butorphanol (Butomidor^®^, Richter Pharma AG, Austria), medetomidine (Domitor^®^, Vetoquinol, France), midazolam (Dormicum, Cheplapharm Arzneimittel GmbH, Germany) and ketamine (Ketamidor^®^, Richter Pharma AG, Austria). They were administered in different dosages, combinations and routes as represented in Table 1.

Once adequate sedation was achieved, both ears were clipped, cleaned and disinfected, and an intravenous (IV) catheter (Introcan, B. Braun, Austria) size 24G or 26G, respectively was placed in the left or right medial or lateral auricular vein. In three rabbits EMLA^®^ cream (Astra Zeneca, Aspen Pharma Trading Limited, Dublin, Ireland) was applied to the skin above the vein prior to insertion of the cannula.

Prior to induction of anaesthesia all rabbits were preoxygenated with 100% oxygen at 2 L/min administered via a tight-fitting face mask. Induction was performed with alfaxalone (Alfaxan ^®^, Jurox Animal Health, Australia) titrated to effect IV. Orotracheal intubation was performed in 13 rabbits, a laryngeal mask was placed in one rabbit, and a tight-fitting face mask was used in two rabbits for administration of inhalant anaesthetics in oxygen. The respective airway device was connected to a non- rebreathing system and anaesthesia was maintained with sevoflurane (Sevoflo^®^, Abbott, United States) or isoflurane (Isoflo^®^, Zoetis, United States) delivered in oxygen. All rabbits were allowed to breathe spontaneously during anaesthesia. If applicable, loco-regional anaesthesia was performed. In case of nociception (defined as an increase in heart rate and IBP > 20% from baseline) a fentanyl (Fentanyl, Janssen, Austria) bolus 2–6 μg kg^−1^ IV was administered as a rescue analgesia.

Depending on the procedure, rabbits were positioned in different positions (Table 1).

The electrocardiogram, end-tidal carbon dioxide tension, body temperature, and arterial oxygen saturation (SpO2) were monitored during anaesthesia using a multiparametric monitor (IntelliVue^®^, Phillips, Germany). Lactated Ringer’s Solution (B. Braun) was infused at 10 mL kg hour^−1^ IV. All parameters were constantly assessed by an experienced anaesthetist and the inspired fraction of volatile anaesthetic was adjusted to maintain adequate anaesthesia.

In case of perianaesthetic complications, appropriate treatment was administered as needed. Episodes of bradycardia were treated with either glycopyrrolate (Glycopyrronium, Accord Healthcare, Austria) 20 μg kg^−1^ IV or atropine (Atropinum sulfuricum, Nycomed Arzneimittel GmbH, Austria) 40 μg kg^−1^ IV, at the discretion of the responsible anaesthetist. Episodes of hypotension were treated with boluses of Ringer’s Solution 5 mL kg^−1^ or Voluven (Volulyte 6%, Fresenius Kabi, Germany) 2 mL kg^−1^ IV.

Postoperatively, medetomidine was reversed in six rabbits with atipamezole (Antisedan^®^, Zoetis, United States). Naloxone (Amomed, Austria) 40 μg kg^−1^ IV was used in one rabbit and flumazenil (Flumazenil Kabi, Fresenius Kabi, Germany) 0.05 mg kg^−1^ IV in eight rabbits. Meloxicam (Metacam ^®^, Boehringer Ingelheim, Germany) was given 1 mg kg^−1^ orally in two rabbits and 0.2–0.5 mg kg^−1^ subcutaneously in eight rabbits. One rabbit (Table 1) was euthanized during the procedure due to poor prognosis.

### 2.2. Blood pressure measurement

#### 2.2.1. Invasive blood pressure

A 24G size catheter was placed in the left or right auricular artery (on the controlateral site of the venous catheter). Ear was extended backwards and care was taken not to bend the artery (a roll of bandage was placed under the ear and the ear was further taped if needed to keep it in position). The arterial catheter was then connected to a disposable pressure transducer (Combitrans, B. Braun, Austria) with an arterial pressure monitoring line. A dynamic pressure response test was performed to detect over- or under-damping of the system prior to the start of the procedure. A fast flush test was subjectively assessed. Quantification of the damping coefficient was not possible in this study. The system was connected to a pressurised bag filled with 0.9% NaCl (B. Braun, Austria) and containing 5 IU mL^−1^ heparin (B. Braun, Germany). The transducer was positioned at the level of the heart, connected to a multiparametric monitor and zeroed to atmospheric pressure before IBP measurements were taken for each new subject. To minimise errors, the IBP monitoring system was carefully checked and periodically flushed to avoid clots and to remove air bubbles that could alter the damping coefficient of the system.

Before the start of each anaesthesia, the accuracy of the pressure transducer was checked with a manometer (mmHg) (VBM Medizintechnik GmbH, Germany). The manometer was previously tested at different pressures in the hypotensive, normotensive and hypertensive range with a mercury column.

#### 2.2.2. PetMAP^™^

The size of the cuff was selected based on the manufacturer’s instructions. The cuff was then snuggly applied distally to the left or right elbow (on the same side as an arterial catheter) and connected to the PetMAP^™^ device. Care was taken to ensure that the cuff was at the level of the heart. If this was not possible, the distance between the cuff and the heart was measured, converted to mmHg (1 cm H2O = 0.73554 mmHg), and the value corrected accordingly.

A single operator was responsible for all measurements. The procedure was the same for each measurement. During the ABP measurement with the PetMAP^™^ (cuff automatically inflated), three IBP values were recorded. The mean of the three IBP values was compared with one value measured with PetMAP^™^. Throughout the procedure, simultaneous measurements of SAP, DAP and MAP were taken every 5 min with the IBP and PetMAP^™^ monitors. A successful measurement was defined as SAP, DAP and MAP values being all displayed on the monitor and the heart rate on the PetMAP^™^ monitor matched the heart rate on the IBP waveform.

### 2.3. Statistics

Statistical analysis was performed on 16 rabbits, comparing IBP and NIBP measurements for SAP, DAP and MAP. To limit the width of the 95% confidence interval for the within-subject population standard deviation to 10%, at least 97 measurements were required for IBP and NIBP. To compare invasive and non-invasive measurements Bland Altman analysis and linear regression were performed with Excel 2016 (Microsoft corporation, Redmond, Washington, United States). Descriptive statistics were performed with SigmaPlot 11.0 (Systat Software, San Jose, California).

## 3. Results

A total of 18 rabbits were included in the study, two were excluded due to failure to place an arterial line or due to life threatening complications, respectively (Table 1). Data collected from 16 rabbits were used for statistical analysis.

Four rabbits developed bradycardia, which was treated with glycopyrrolate (two rabbits) or atropine (two rabbits). Hypotension (defined as a MAP <60 mmHg) developed in five rabbits and was treated with boluses of Lactated Ringer’s Solution (three rabbits) or Voluven (two rabbits). Additional analgesia (fentanyl bolus) was required in seven rabbits.

Bland–Altman analysis included 158, 154, and 155 comparative IBP-NIBP measurements for MAP, DAP, and SAP, respectively. The number of measurements per rabbit ranged from 4 to 23 with a median of 10 [8–12 (25–75%)]. The number of successful measurements for each pressure and measurement technique is shown in Table 2.

**Table 2:** Number of measurements for SAP, DAP and MAP for IBP and PetMAP^™^ measuring techniques.

IBP			PetMAP^™^		
SAP	DAP	MAP	SAP	DAP	MAP
158	158	164	166	165	163
92.9%	92.9%	96.5%	97.6%	97.1%	95.9%

IBP, Invasive blood pressure; SAP, Systolic arterial pressure; DAP, Diastolic arterial pressure; MAP, Mean arterial pressure.

The percentages represent percentage of successful measurements. Lower percentage of IBP results in the fact, that these values represent an average of three measurements. If one measurement was not valid, data was excluded.

**Figure 1:**
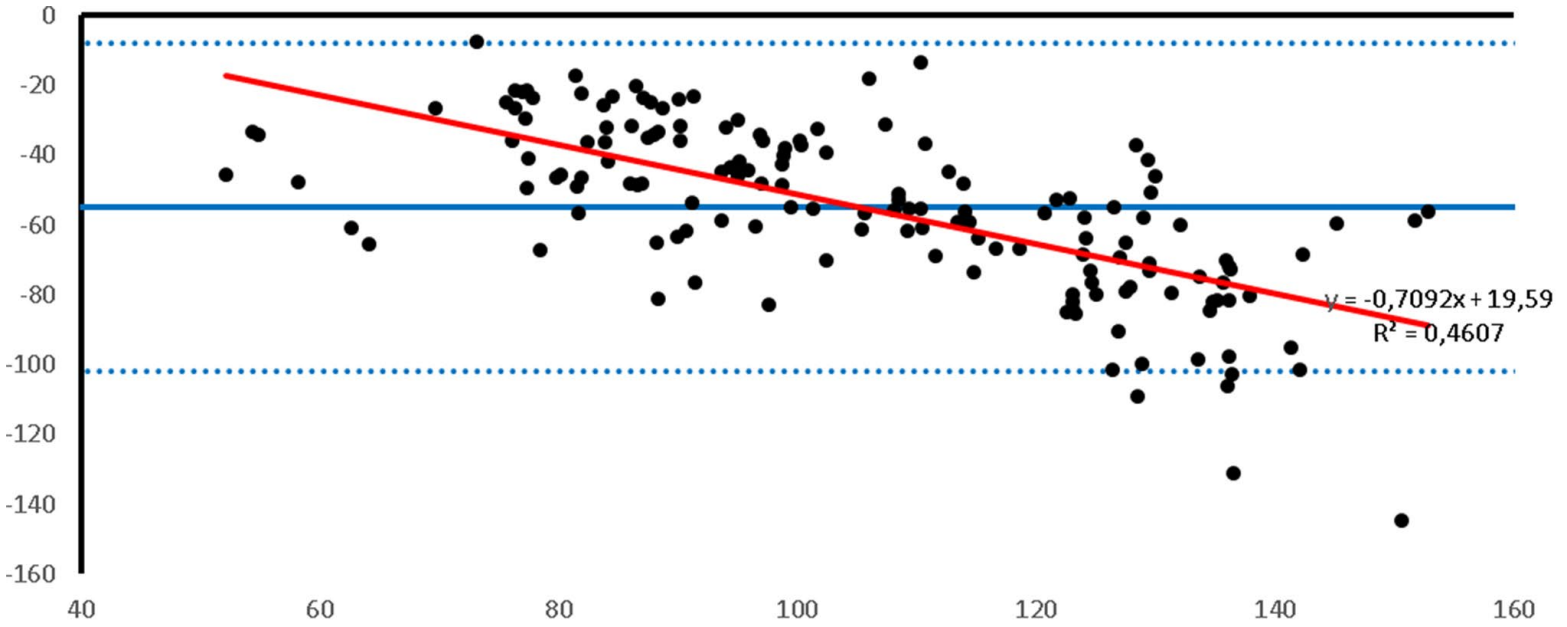
Bland–Altman plot of the agreement between the invasive SAP and the SAP measured with the PetMAP^™^ device. Mean value of PetMAP^™^ and IBP measurement is presented on the *X* axis [(PetMAP^™^ + IBP)/2] and the difference between IBP and PetMAP^™^ measurement on the *Y* axis (IBP-PetMAP^™^). Solid line indicates bias, dashed lines indicate upper and lower limits of agreement, and red solid line indicates linear regression.

**Figure 2:**
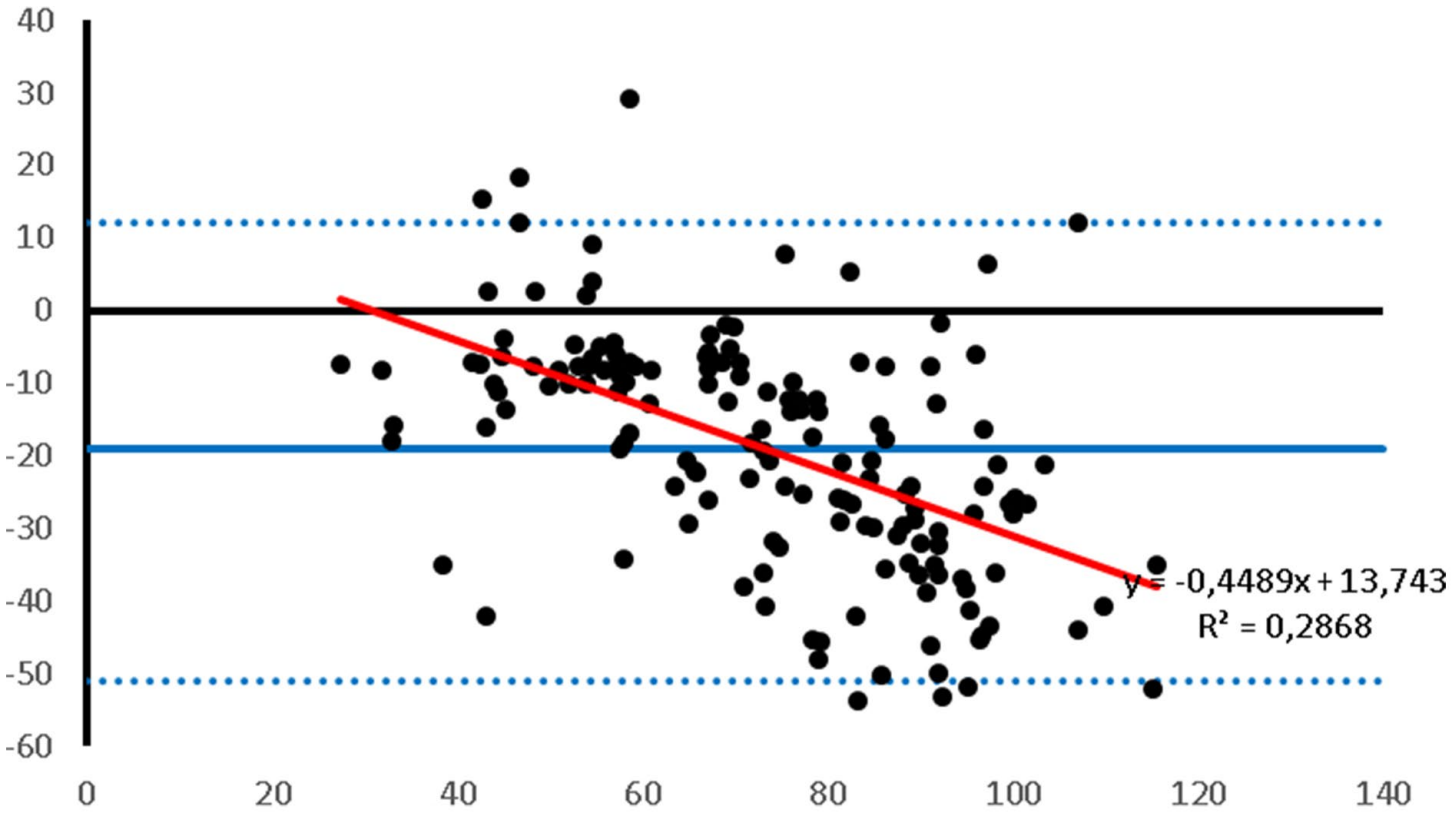
Bland–Altman plot of the agreement between the invasive DAP and the DAP measured with the PetMAP^™^ device. Mean value of PetMAP^™^ and IBP measurement is presented on the *X* axis [(PetMAP^™^ + IBP)/2] and the difference between IBP and PetMAP^™^ measurement on the *Y* axis (IBP-PetMAP^™^). Solid line indicates bias, dashed lines indicate upper and lower limits of agreement, and red solid line indicates linear regression.

**Figure 3:**
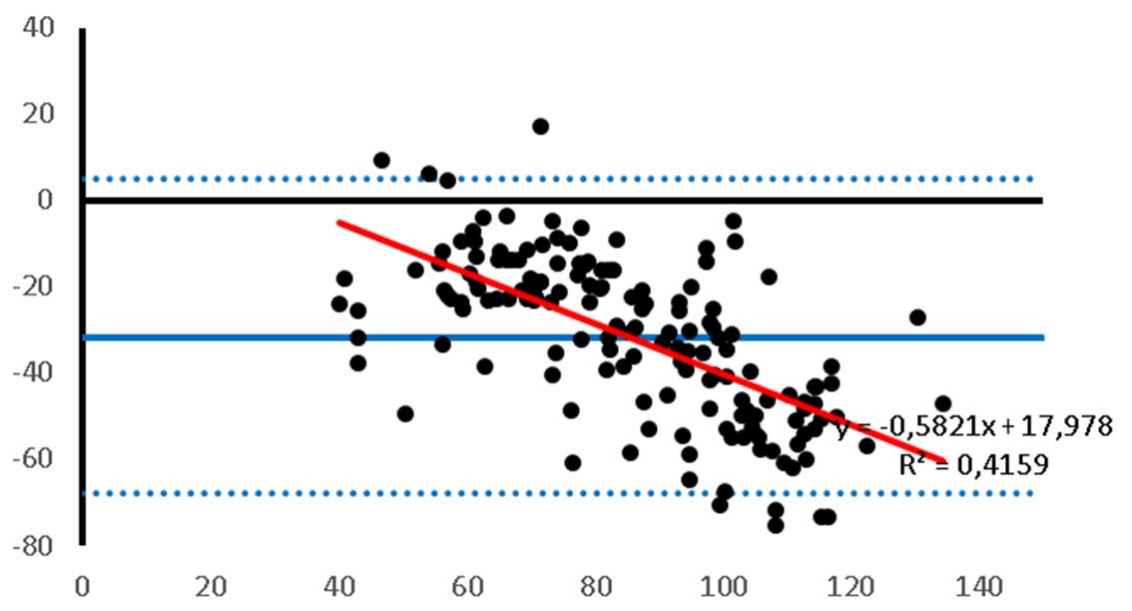
Bland–Altman plot of the agreement between the invasive MAP and the MAP measured with the PetMAP^™^ device. Mean value of PetMAP^™^ and IBP measurement is presented on the *X* axis [(PetMAP^™^ + IBP)/2] and the difference between IBP and PetMAP^™^ measurement on the Y axis (IBP-PetMAP^™^). Solid line indicates bias, dashed lines indicate upper and lower limits of agreement, and red solid line indicates linear regression.

Bland–Altman plots showed that the PetMAP^™^ device overestimated the IBP measurements of SAP, DAP and MAP which can be seen from the bias (Figures 1–3). The mean bias was −55, −19 and − 32 mmHg for SAP, DAP and MAP, respectively. The mean difference of paired measurements of SAP, DAP and MAP for the PetMAP^™^ device and the invasive method compared with the recommendations of ACVIM is shown in Table 3. The PetMAP^™^ device did not meet any of the ACVIM.

**Table 3:** The agreement of SAP, DAP, and MAP measurements between the PetMAPTM device and the IBP measurement method compared with the ACVIM recommendations for validation of NIBP measurement devices.

Parameter	ACVIM recommendations	SAP	DAP	MAP
Mean bias (mmHg) †	−10 mmHg ≤ bias ≤10 mmHg	**−55**	**−19**	**−32**
SD (mmHg)	≤15	**24**	**16**	**19**
≤ ±5 mmHg (%) ‡	NS	**0**	**7.8**	**3.2**
≤ ±10 mmHg (%)‡	≥50	**0.6**	**33.1**	**9.5**
≤ ±20 mmHg (%)‡	≥80	**2.6**	**53.2**	**30.4**

ACVIM, American College of Veterinary Internal Medicine; SAP, Systolic arterial pressure; DAP, Diastolic arterial pressure; MAP, Mean arterial pressure; NS, Not specified; SD, Standard deviation; †Mean bias: average of all differences (IBP – PetMAP^™^), ‡Percentages of PetMAP^™^ measurements (SAP, DAP, MAP) lying within ≤ ±5, 10 or 20 mmHg of the corresponding IBP values.

Figures 1–3 show Bland–Altman analysis results with bias, upper and lower limits of agreement between SAP, DAP, and MAP, respectively, which were measured invasively in the auricular artery and with a PetMAP^™^ device.

## 4. Discussion

In the present study the ABP measurements taken by PetMAP^™^ and IBP measured in the auricular artery in rabbits undergoing general anaesthesia showed poor agreement. More specifically, PetMAP^™^ significantly overestimated invasively measured SAP, DAP and MAP. The agreement between the two methods was the poorest for SAP where PetMAP^™^ overestimated IBP by up to 145 mmHg (average bias −55 mmHg). The best agreement in between the two devices was found for DAP, however PetMAP^™^ still overestimated invasive DAP by up to 54 mmHg (average bias −19 mmHg). As linear regression shows, an overestimation increased with higher pressures for SAP, DAP and MAP.

In rabbits, different oscillometric ABP measurement devices have already been compared to IBP measurements – with contrasting results (10–13). A study from 2005 (10) demonstrated moderate or poor accuracy of measurements when the cuff was applied to the anterior or posterior limb, respectively. Another study (11) suggested oscillometric ABP measurements as a poor substitute for IBP measurements. On the other hand some authors (12) demonstrated acceptable agreement between oscillometric MAP and DAP measurements and the invasive technique based on ACVIM guidelines. In 2021 (13) a high definition oscillometric device was tested and good agreement with IBP measurements when the cuff was applied distal to the elbow was found. Similar as in our study some other authors (11–13) found the poorest agreement between NIBP and IBP mesurement techniques for the SAP.

The ACVIM has published recommendations for the validation of noninvasive blood pressure monitoring devices (3). The recommendations are based on the more stringent human medical guidelines of the AAMI. For the device to be validated according to the ACVIM recommendations, the mean bias must be no more than +/− 10 mmHg, with a standard deviation of no more than 15 mmHg, and 50 and 80% of all measurements must be within 10 mmHg and 20 mmHg of the reference method, respectively (3). In rabbits the PetMAP device did not meet any of the ACVIM recommendations.

This result is surprising, since various authors have demonstrated good – acceptable agreement in between IBP and PetMAP^™^. In anesthetised cats, regardless of cuff position, PetMAP^™^ met the AAMI criteria for agreement with IBP (9). In normotensive dogs, various authors have shown good agreement between PetMAP^™^ and IBP, with a tendency of PetMAP^™^ to underestimate the IBP. In general, MAP and DAP were predicted more reliably and with a higher accuracy than SAP (8, 14).

A possible explanation for these contrary observations is that the PetMAP^™^ device has not been designed for rabbits, but specifically for cats and dogs. This explanation is supported by the results of other authors (7), who used the PetMAP^™^ device in sheep anaesthetized for ovariohysterectomy. These authors observed an underestimation of IBP pressures with PetMAP^™^, resulting in an inability of PetMAP^™^ to meet the AAMI guidelines. However, most of the ACVIM criteria were met and the authors suggested that the accuracy of PetMAP^™^ may increase in hypotensive states.

In fact, the manufacturer states that it may be used in other species given that the cuff can be properly positioned on a limb. For the rabbits in the present study the cuff was placed on an anterior limb. According to the manufacturer’s instructions a non-optimised measurement mode was selected (a mode used for species other than cats and dogs). The choice of the anterior limb as the site of cuff placement was based on previously published data showing that the position of the NIBP cuff on an anterior limb resulted in better agreement between noninvasive and invasive blood pressure measurements (10, 13).

Still, the shape of the rabbit’s limbs may have affected the measurements. Sphygmomanometric techniques for measuring ABP work by occluding a bigger superficial artery with inflation of a suitable cuff. Therefore, an unsuitable limb shape could hinder the compression of the artery when using a standard cuff (15). This may explain the poor agreement in between the devices in this study if the PetMAP cuff may have had difficulties properly occluding the underlying artery. Another possible reason may be the small size of arteries in rabbit limbs, resulting in comparably weak oscillations which are difficult to detect by an oscillometric device. This explanation is supported by the findings of other authors (15–17) who reported difficulties in obtaining ABP readings with an oscillometric device in smaller patients.

Interestingly, in hypotensive dogs PetMAP^™^ tended to greatly overestimate the IBP measurements (18), similar to the results observed in this study. Rabbits are prone to hypotension during anaesthesia, with 92% of rabbits showing SAP or MAP pressures below 80 or 60 mmHg, respectively at surgical planes of anaesthesia (19). This complication was also observed in the rabbits in this study, which may explain the overestimation of IBP readings by PetMAP^™^. Unfortunately, since the PetMAP^™^ device in the present study did not accurately predict the changes in invasive auricular pressure, the correction factors described by other authors (11, 20) could not be directly applied for all ranges of pressures. Further studies attempting to identify a correction factor that can be applied to PetMAP^™^ measurements to approximate IBP measurements are certainly required.

Invasive blood pressure measurements are considered a gold standard but can also be a source of error (5, 21). In a study from 2020 (21) it was shown that the IBP measurement system is more prone to error with higher compared to lower heart rates and that at high heart rates (120 beats per minute) the demands on the system become more stringent. Heart rates in rabbits are usually higher than 120 beats per minute, even when cardiodepressive drugs have been used for sedation or anaesthesia (22). This could have influenced the accuracy of the invasive measurement. Furthermore, catheters placed in an artery in rabbits are small and could be more prone to kinking or occlusion (clots, pressing on the vessel wall, positioning of the ear). In the present study Emla (Emla^®^, AstraZeneca, United Kingdom) cream was used in some of the rabbits. In humans it was demonstrated that Emla cream produces a biphasic vascular response (23). It is therefore possible that it affected vascular tone in rabbits as well and had an effect on IBP measurements. Incorrect IBP measurements would therefore inadequately reflect the agreement with the PetMAP device.

This study has several limitations.

For once, IBP was measured in a peripheral artery as compared to a central one. This decision was based on a risk–benefit evaluation for the clinical patient. While the more invasive catherterisation of a central artery was not considered appropriate, this choice may have affected the IBP measurements. Several authors (10, 11, 20) have examined the agreement of invasive pressure measured in the auricular artery with pressure measured in the more central arteries in rabbits and found different results. Some authors (10) reported a strong correlation between measurements in the central auricular artery and measurements in the abdominal aorta (via femoral artery catheterization). Contrarily, others (11, 20) reported a greater discrepancy between invasive pressure measurement sites, with SAP and MAP being underestimated and DAP overestimated when measured in the auricular artery compared with the carotid artery. However, if the findings from the latter studies would be taken into account in the present study, the differences between the DAP measured invasively in the auricular artery and with PetMAP^™^ would be even greater. And even while the agreement for SAP and MAP between the both methods would be better, PetMAP^™^ would still not meet the recommendations for validation of NIBP measurement devices.

Another possible limitation was the selection of clinical patients. Rabbits in this study varied in size, age and ASA status and anaesthetic protocols as well as positioning of the patients differed. In rabbits, the effect of different anaesthetic protocols on the agreement between high definition oscillometric device and invasive blood pressure measurements was examined by others (13) and the authors suggested that anaesthesia-related differences in vascular resistance influenced the accuracy of the noninvasive blood pressure measurement device. In this study, several different anaesthetic protocols were used which could have affected the accuracy of the blood pressure monitors. However, this variety of protocols reflects the clinical practice, where anaesthetic protocols are individually selected based on the health status of the animal and the requirements of the procedure. Similarily, while it has been shown that the body position may have a significant effect on NIBP measurements in awake dogs (24), in a clinical situation, body position during anaesthesia cannot be freely chosen but is adjusted to the requirements of the procedure.

Finally, the damping coeficient was not determined in the present study. Overdamping or underdamping in a pressure system will result in falsely low or falsely high IBP readings (25). Because the damping coefficient was not measured, an accurate estimate of overdamping or underdamping (and thus an assessment of the accuracy of the IBP measurements) was not possible in the present study.

## 5. Conclusion

In the clinical setting, the PetMAP^™^ device showed significant overestimation of SAP, DAP, and MAP, which were measured in the auricular artery. In addition, the bias was not constant, implying that the device poorly predicted changes in blood pressure. The PetMAP^™^ device did not meet any of the ACVIM recommendations.

## Data availability statement

The raw data supporting the conclusions of this article will be made available by the authors, without undue reservation.

## Ethics statement

The animal study was reviewed and approved by the Institutional Ethics and Animal Welfare Committee of the University of Veterinary Medicine Vienna. Written informed consent was obtained from the owners for the participation of their animals in this study.

## Author contributions

JS, AR, and N-GK prepared study design and were involved in clinical management of the cases. AR and N-GK supervised the experiment. JŽ performed statistical analysis. All authors reviewed and edited the manuscript, have read, and approved the final manuscript.

## Conflict of interest

The authors declare that the research was conducted in the absence of any commercial or financial relationships that could be construed as a potential conflict of interest.

## Publisher’s note

All claims expressed in this article are solely those of the authors and do not necessarily represent those of their affiliated organizations, or those of the publisher, the editors and the reviewers. Any product that may be evaluated in this article, or claim that may be made by its manufacturer, is not guaranteed or endorsed by the publisher.
